# Cancer-related identity and advanced old age – analysis of prostate cancer survivors after radical prostatectomy over the age of 75

**DOI:** 10.1186/s12877-025-06477-5

**Published:** 2025-10-06

**Authors:** Matthias Jahnen, Charlotte Bierwirth, Valentin H. Meissner, Andreas Dinkel, Stefan Schiele, Helga Schulwitz, Jürgen E. Gschwend, Kathleen Herkommer

**Affiliations:** 1 Department of Urology, School of Medicine and Health, TUM University Hospital, Technical University of Munich, Ismaninger Str. 22, Munich, 81675 Germany; 2 Department of Psychosomatic Medicine and Psychotherapy, School of Medicine and Health, TUM University Hospital, Technical University of Munich, Langerstr.3, Munich, 81675 Germany

**Keywords:** Prostate cancer, Cancer survivors, Aging, Cancer-related identity, Cancer-related self-perception, Comorbidities

## Abstract

**Background:**

The struggle to incorporate the experience of suffering from PCa in their biography can be evident in men affected by PCa even years after the initial cancer diagnosis. Aim of this study was to assess the cancer-related identity and associated factors in men over 75 years affected by PCa and treated with radical prostatectomy to improve the understanding of cancer-related self-perception in older men.

**Methods:**

2,379 on average 82.2 ± 3.9 year-old prostate cancer survivors with a mean follow-up of 17.9 ± 3.7 after radical prostatectomy were asked to choose one of 5 cancer-related identities (“patient”, “victim”, “someone who has had cancer”, “cancer survivor,” and “cancer conqueror”). Associations with clinical data, aging-related factors (frailty, comorbidities, loneliness), and psychological factors were assessed.

**Results:**

Most men identified with the neutral terms “someone who has had cancer” (45.7%) and patient (27.0%), which was associated with primarily clinical characteristics. Identification with negatively connoted cancer-related identities was less common (“cancer survivor” (15.7%), “victim” (2.1%)) and was associated with primarily aging-related and psychological factors. Identification as a “cancer survivor” was associated with more self-reported comorbidities (OR: 1.09 [1.01 – 1.17]) and symptoms of depression (OR: 1.71 [1.20 – 2.45]). Identification as a victim was associated with severe loneliness (OR: 5.09 [2.39 – 10.85]). The positively connoted cancer-related identity “cancer conqueror” (9.5%) was associated with a higher quality of life (OR: 1.41 [1.28 – 1.57]).

**Conclusions:**

The cancer-related identity in long-term PCa survivors of advanced old age varies widely. Especially negatively connoted cancer-related identities reflect the subjective disease experience, the overall health status, and the lack of a social support system. Even years after the primary diagnosis and treatment, assessment of the cancer-related identity can identify men who are affected most by their cancer experience and might need further psycho-oncological assistance.

## Background

Prostate cancer (PCa) is one of the most often diagnosed malignant diseases in the world [1]. Early detection and various treatment options allow long-term survival after the initial diagnosis in most men affected by PCa. Men can often be cured or live with the disease for many years [2]. This has led to an increasing group of men of advanced old age affected by PCa and PCa treatment such as radical prostatectomy [3]. Although the excellent long-term survival in these men might suggest that the significance of PCa decreases with increasing age, fear of symptomatic progression or death caused by PCa and lingering therapy side effects concern many of these men [4]. Much research has been conducted regarding psychological adaptation and needs in the early years after PCa diagnosis and initial therapy [5, 6]. However, research on older men beyond 75 years of age who have survived PCa for many years remains sparse.

Nevertheless, it has been shown that cancer-related distress, fear of progression, and the struggle to incorporate the experience of suffering from PCa in their biography can be evident in men affected by PCa even years after the initial cancer diagnosis [7–9]. In this regard, it has repeatedly been shown that assessment of the cancer-related self-perception or cancer-related identity helps distinguish men profoundly affected by their PCa experience and may have the most significant need for further psycho-oncological support [10–13]. Men who identify more negatively with their cancer experience seem to have a significantly lower quality of life and higher cancer-related distress than men with a neutral or even positive cancer-related self-perception [5, 10, 14]. Therefore, it seems valuable to identify variables that might influence the development of a specific cancer-related identity. It has been shown that some clinical variables that reflect the intensity of the PCa treatment and the disease course seem to be linked to specific cancer-related identities. A more demanding cancer experience appears to be associated with a more loaded cancer-related identity [10, 12, 15, 16].

In contrast, an uneventful cancer experience seems to be associated with a balanced cancer-related identity [5, 10, 11]. However, objective clinical characteristics play a minor role in developing a cancer-related identity compared to subjective disease-related characteristics such as the severity of the disease. Moreover, whether PCa-unrelated health factors contribute to specific cancer-related identities is unclear. Especially in older patients, comorbid somatic disorders, signs of aging, and changing life priorities may influence one’s self-perception regarding PCa. Old age is often accompanied by physical decline [17, 18], increasing frailty [19], the loss of friends and family, and increasing loneliness and solitude [17–19]. However, data on how these potential ramifications of aging might influence cancer-related self-perception has yet to be investigated.

Therefore, we aimed in this analysis to assess the cancer-related identity in a large sample of 2,379 men over 75 years who have been affected by PCa and treated with radical prostatectomy. Further, we assessed associations between cancer-related identities and a broad set of characteristics reflecting these men’s subjective health status, quality of life, loneliness, and frailty.

## Methods

### Design and procedure

The data were obtained from the ongoing German research project “Familial Prostate Cancer”. Since 1999, this research project has been recruiting men from across Germany who have undergone treatment for prostate cancer at various medical facilities. Baseline sociodemographic and clinical data are collected at the time of study entry and updated as part of an annual questionnaire. This yearly questionnaire is identical for all participating men and contains items assessing various variables each year, allowing for the examination of different aspects of prostate cancer survivorship. Additional clinical data are obtained from the treating urologist. The objective of this study is to evaluate the long-term oncological, psychological, and functional outcomes in men who have undergone treatment for localized prostate cancer. A full account of the details of the research project has been provided elsewhere. In the present study, cross-sectional data from the annual follow-up conducted in 2022 were analyzed. By January 2023, 3,086 of the 4,476 participants (68.9%) had returned the questionnaire. Participants aged 75 years or older who underwent radical prostatectomy as primary treatment and answered the item regarding cancer-related identity were included in the analysis (*N* = 2,379, 77.1%). The ethics committee of the Technical University of Munich has approved this research project (Ref. No. 25/20S).

### Measures

#### Cancer-related identity

Following previous research, the participants were asked to choose which of the following terms they considered most suitable to describe their personal cancer experience: “patient” (German translation: “Patient”), “victim” (“Opfer”), “someone who has had cancer” (“Jemand der Krebs hatte”), “cancer survivor” (“Krebsüberlebender”) and “cancer conqueror” (”Krebsbezwinger”) [11, 14].

#### Sociodemographic and clinical characteristics

The following sociodemographic data were included in this analysis: age at the survey, school education, subjective financial situation, current partnership, and children. Clinical data included were: time since surgery, presence of a second primary cancer, family history of PCa (yes: at least one consanguine relative with PCa vs. no), biochemical recurrence (PSA level ≥ 0.2 ng/ml) during follow-up, biochemical recurrence at survey, and ongoing PCa treatment at survey.

#### Depression and anxiety

Using the validated ultra-brief instruments Patient Health Questionnaire-2 (PHQ-2) and General Anxiety Disorder-2 (GAD-2) scale, symptoms of depression and anxiety were assessed. For both scales (range 0–6), a cut-off score ≥ 3 is an indication of the presence of clinically significant depression or anxiety, respectively [20, 21]. Cronbach’s alpha coefficients for PHQ-2 and GAD-2 scale were 0.74 and 0.79, respectively.

#### Global health status/quality of life

Quality of life was assessed using the last two items of the European Organization for Research and Treatment of Cancer questionnaire (EORTC QLQ-C30), capturing the overall health and quality of life in the past week. Answers were given on a seven-point Likert scale ranging from `very poor´ [1] to ‘excellent’ [7]. Following the standardized EORTC formula, the mean value of the two items ranged from 0 to 1. A higher score suggests a higher quality of life [22]. 

#### Comorbidities

The Self-Administered Comorbidity Questionnaire (SCQ) assessed the total number of perceived comorbidities. The questionnaire contains a list of 13 common health problems (including cancer) and medical conditions. Participants indicate the frequency of certain medical conditions (`number of comorbidities´), and whether these medical conditions are “a problem” for the participant (`total number of burdening comorbidities´). Additionally, choosing cancer as a comorbidity was assessed as an independent item.

#### Loneliness

Loneliness was assessed using a single question: “I am frequently alone/have few contacts.” Participants could choose one of five response options: [0] No, does not apply; [1] Yes, it applies, but I do not suffer from it; [2] yes, it applies, and I suffer slightly; [3] yes, it applies, and I suffer moderately, and [5] yes, it applies, and I suffer strongly. Loneliness was stratified by combining [0] and [1]: `no (loneliness),´ [2]: `yes, mild (loneliness),´ and combining [3] and [4]: `yes, severe (loneliness).´ [23].

#### Frailty

Frailty was assessed using the 15-item Groningen Frailty Index, which covers four domains of functionality, taking into account physical and cognitive limitations and emotional and psychosocial aspects. Each of the 15 items may be answered with either yes [1] or no (0). The answers to all items were summed up, and a score of ≥ 4 indicated clinically significant frailty [24].

### Statistical analysis

Descriptive statistics were calculated for all study variables. Using Chi-square and Wilcoxon tests, differences between each of the 5 cancer-related identities were analyzed concerning the sociodemographic, clinical, and psychological variables. Further 5 separate multivariable logistic regression analyses with backward elimination were used to identify variables independently associated with each of the five cancer-related identities. Significance was set at *p* < 0.05. All analyses were performed using SAS (Version 9.4).

## Results

**Fig. 1:**
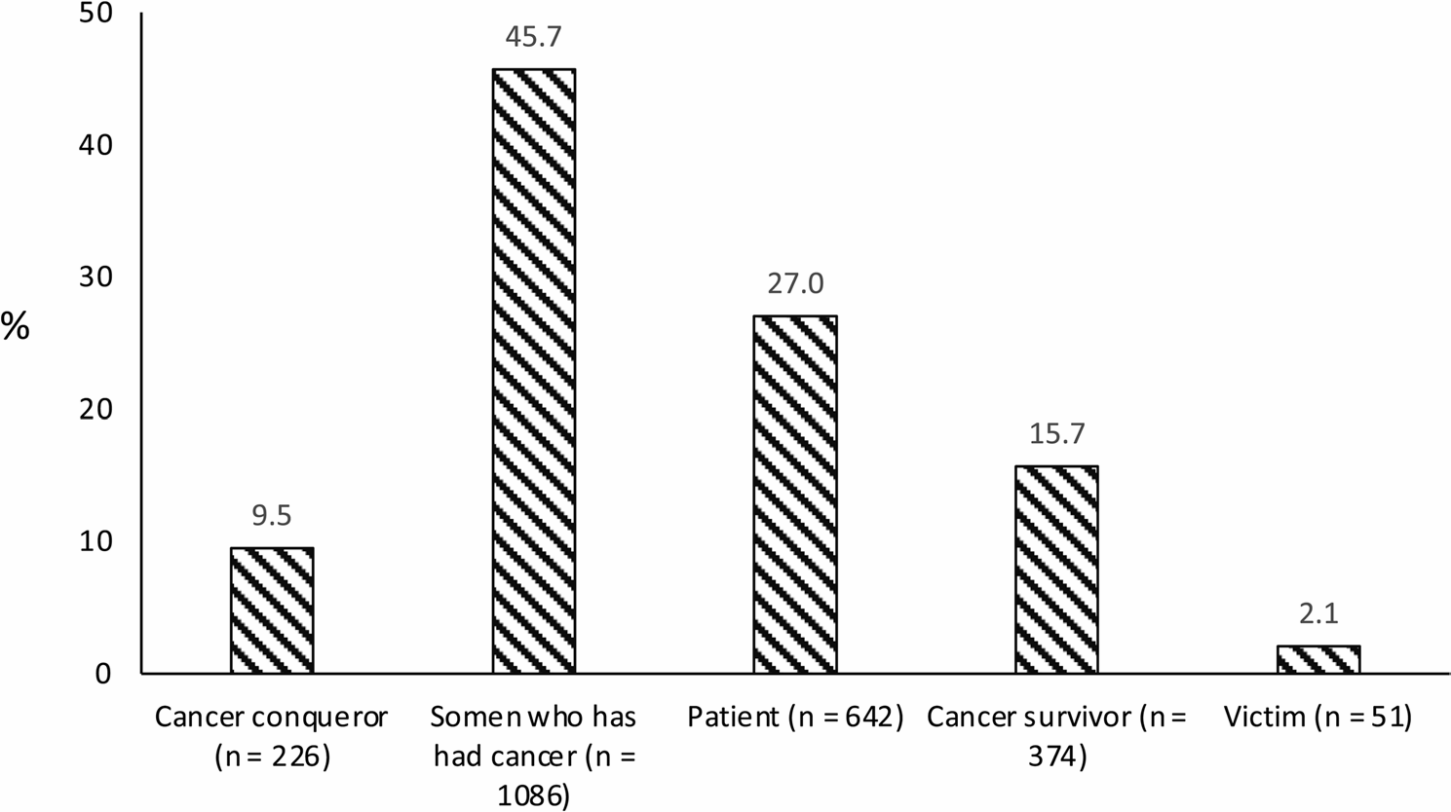
Cancer-related identity in the study sample of men affected by prostate cancer with a median follow-up of 17.4 years

2,379 men affected by PCa with a mean age of 82.2 ± 3.9 years at the survey and a mean follow-up of 17.9 ± 3.7 years since prostatectomy were included in the analysis (Table 1). Men identified most often as “someone who has had cancer” (45.7%; 1,086/2,379) and “patient” (27.0%; 642/2,379). 15.7% (374/2,379) identified as “cancer survivors” and 9.5% (226/2,379) as “cancer conquerors”. “Victim” was the least endorsed self-description (2.1%; 51/2,379) (Fig. 1).

**Table 1:** Sociodemographic, clinical and psychological characteristics of the study sample (*N* = 2,379)

	*n*	%
Sociodemographic characteristics		
*Age at survey (years)Mean (SD):82*.2 (3.9)		
75 - < 80	727	30.6
≥ 80 - < 85	1104	46.4
≥ 85	548	23.0
*Educational level*		
primary, secondary - low	920	40.3
secondary - intermediate	360	15.8
secondary - high	290	12.7
tertiary	715	31.3
*Financial situation*		
very bad	5	0.2
bad	29	1.2
satisfactory	622	26.5
good	1341	57.0
very good	355	15.1
*Partnership*		
yes	2623	86.6
no	405	13.4
*Children*		
0	254	10.8
≥ 1	2092	89.2
Clinical characteristics		
*Time since surgery (years) Mean (SD)*: 17.9 (3.7)		
≤ 10	24	1.0
> 10 ≤ 15	480	20.2
> 15 ≤ 20	1215	51.1
> 20	660	27.7
*Second primary cancer*		
yes	345	14.5
no	2034	85.5
*Family history of PCa*		
yes	885	37.2
no	1494	62.8
*Biochemical recurrence*		
yes during follow-up	439	18.5
yes at survey	443	18.6
no	1497	62.9
*Ongoing treatment at survey*		
yes	253	10.6
no	2126	89.4
*Total number of subjective comorbidities SCQ* Mean (SD): 3.04 (1.85)		
*Cancer a subjective comorbidities*		
yes	1961	82.4
no	418	17.6
*Subjective urological comorbidities*		
yes	151	6.3
no	2228	93.7
*Frailty GFI-Score Mean (SD) 3.04 (2.5)*		
negative (< 4)	1433	64.6
positive (≥ 4)	785	35.4
Psychosocial factors		
* PHQ-2 (symptoms of depression)*		
no (< 3)	2031	89.0
yes (≥ 3)	252	11.0
*GAD-2 (symptoms of anxiety disorder)*		
no (< 3)	2074	91.4
yes (≥ 3)	195	8.6
*Quality of Life* Mean (SD): 0.66 (0.19)		
* Loneliness*		
yes, severe	151	6.5
yes, mild	241	10.4
no	1937	83.2

*M* Mean, *SD* Standard deviation, *PCa* prostate cancer, *PHQ* Patient health questionnaire, *GAD* General anxiety disorder, *SCQ* Self-Administered Comorbidity Questionnaire

**Fig. 2:**
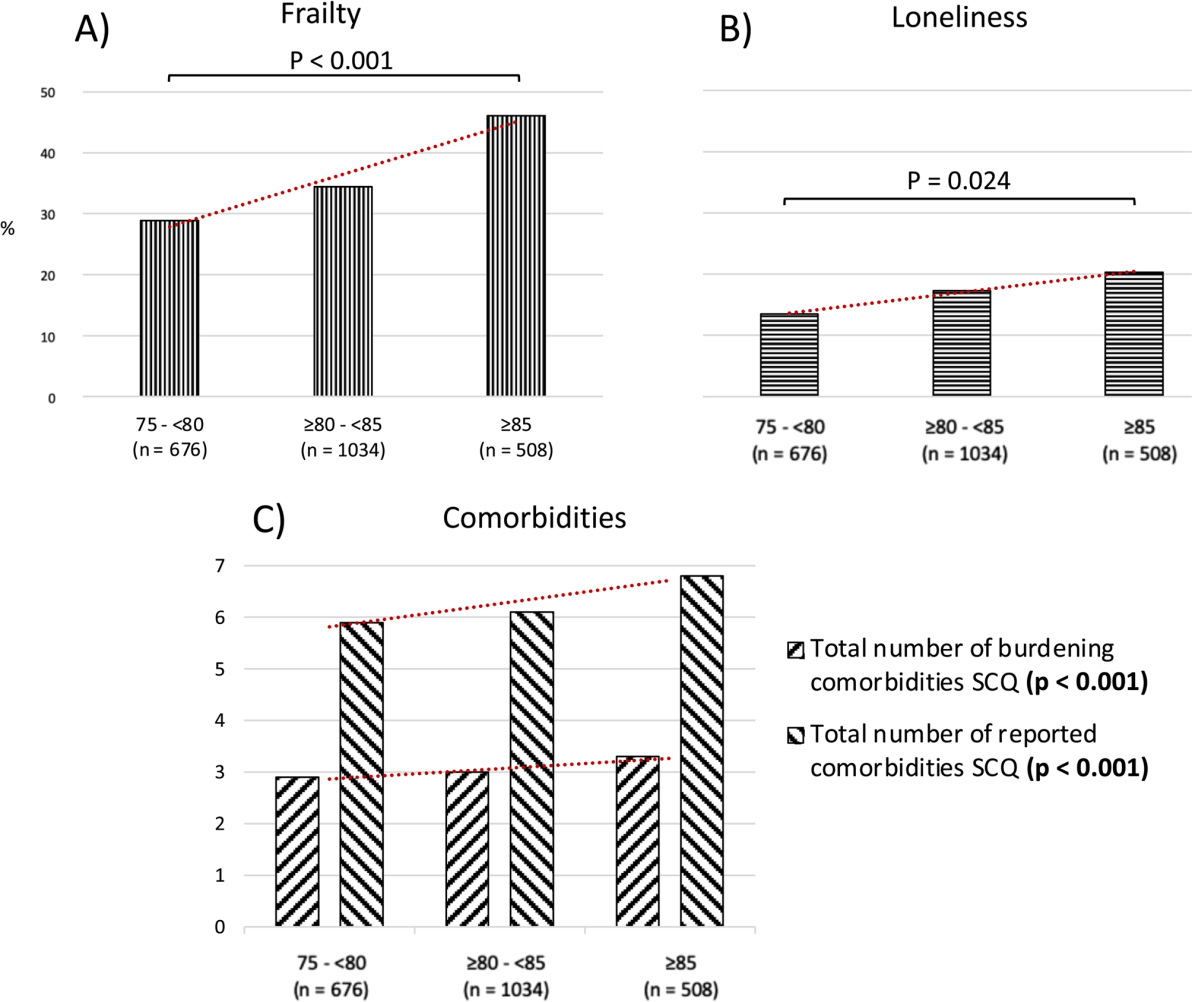
Percentage of men reporting frailty **A**, loneliness (mild plus severe) **B** and total number of subjective comorbidities and burdening subjective comorbidities **C** stratified by age groups

Frailty, loneliness, and the number of subjective comorbidities were highest in men > 85 years of age, compared to men aged 75–80 and 80–85 (all *p* < 0.05; Fig. 2).

Men identifying as “cancer survivor” or “victim” stated more often to have a less than satisfactory financial situation (2.2% (cancer survivor) and 10.0% (victim), respectively vs. 1.1% (rest of the sample); *p* < 0.001) and were less often in a partnership (81.5% (cancer survivor) and 74.0% (victim), respectively vs. 86.1% (rest of the sample); *p* = 0.017). Men identifying as “someone who has had cancer” or “patient” reported more often a higher educational level (32.3% (someone who has had cancer) and 36.7% (patient), respectively vs. 24.6% (rest of the sample); *p* < 0.001). (Table 2)

**Table 2:** Comparison of key characteristics of the 5 cancer-related identities using Chi-square and Wilcoxon tests

	*Someone who has had cancer* *n = 1086*	Patient *n* = 642	*Cancer conqueror* *n = 226*	Cancer survivor *n* = 374	Victim *n* = 51
Total number of reported comorbidities SCQ *** M(SD)	6.1(4.3)	5.9(4.1)	5.4(4.2)	7.1(4.6)	6.7(4.5)
Total number of burdening comorbidities SCQ *** M(SD)	3.0(1.9)	2.9(1.8)	2.7(1.8)	3.5(1.9)	3.4(1.9)
Cancer a subjective comorbidities*** (%)					
* yes*	12.6	23.4	14.2	24.6	13.7
* no*	87.4	76.6	85.8	75.4	86.3
*Subjective urological comorbidities (%)*					
* yes*	6.3	6.1	4.0	8.3	5.9
* no*	93.7	93.9	96.0	91.7	94.1
Frailty*** (%)					
* yes*	34.5	33.5	26.8	44.9	45.6
* no*	65.5	66.5	73.2	55.1	54.4
Loneliness*** (%)					
yes, severe	5.8	4.9	4.5	9.6	26.0
yes, mild	10.4	7.8	10.9	14.5	8.0
no	83.8	87.3	84.6	75.9	66.0
Age at survey (years) ** (%) M(SD)	82.0 (3.9)	82.2 (4.0)	82.2 (3.7)	82.4 (4.1)	82.4 (3.2)
75 - <80	31.8	31.2	28.3	28.6	21.6
≥ 80 - <85	46.2	44.5	50.9	46.0	56.9
≥ 85	22.0	24.3	20.8	25.4	21.6
Educational level *** (%)					
primary, secondary - low	39.3	34.6	47.7	46.4	54.2
secondary - intermediate	16.4	14.9	14.7	15.3	20.8
secondary - high	12.0	13.9	11.5	13.1	16.7
tertiary	32.3	36.7	26.1	25.3	8.3
Financial situation*** (%)					
very poor/poor	0.9	1.1	1.8	2.2	10.0
satisfactory/good/very good	99.1	98.9	98.2	97.8	90.0
Partnership * (%)					
yes	84.8	88.3	85.8	81.5	74.0
no	15.2	11.7	14.2	18.5	26.0
*Children (%)*					
0	10.6	10.3	10.8	12.2	13.7
≥ 1	89.4	88.7	89.2	87.8	86.3
Time since surgery (years)*** (%)					
≤ 10	0.8	1.5	0.4	1.1	0.0
> 10 ≤ 15	21.0	20.4	19.0	16.8	29.4
> 15 ≤ 20	52.8	53.0	50.9	45.2	35.3
> 20	25.4	25.1	29.7	36.9	35.3
*Second primary cancer (%)*					
yes	14.2	14.3	15.9	15.2	11.8
no	85.8	85.7	84.1	84.8	88.2
*Family history of PCa (%)*					
yes	36.7	38.0	35.4	38.5	37.3
no	63.3	62.0	64.6	61.5	62.7
*Biochemical recurrence (%)*					
yes during follow-up	16.8	16.8	23.5	23.8	11.8
yes at survey	15.1	23.4	15.5	22.2	21.6
no	68.1	59.8	61.0	54.0	66.7
Ongoing treatment at survey*** (%)					
yes	6.1	16.0	9.7	15.5	7.8
no	93.9	84.0	90.3	84.5	92.2
PHQ-2 (depression screening)*** (%)					
negative (< 3)	90.9	90.5	87.7	82.6	78.7
positive (≥ 3)	9.1	9.5	12.3	17.4	21.3
GAD-2 (anxiety disorder screening)*** (%)					
negative (< 3)	93.1	92.4	92.1	85.9	78.3
positive (≥ 3)	6.9	7.6	7.9	14.1	21.7
Quality of Life*** M(SD)	0.66(0.18)	0.66(0.19)	0.73(0.20)	0.62(0.19)	0.55(0.19)

*M* mean, *SD* Standard deviation, *PCa* Prostate cancer, *PHQ* Patient health questionnaire, *GAD* General anxiety disorder, *SCQ* Self-Administered Comorbidity Questionnaire

^*^p-value < 0.05

^**^p-value < 0.01

^***^p-value < 0.001

An ongoing PCa treatment was most common in men identifying as “patient” and “cancer survivor” (16.0% (patient) and 15.5% (cancer survivor), respectively vs. 6.7% (rest of the sample); *p* < 0.001). These men also acknowledged “cancer” most often as a subjective health problem (23.4%(patient) and 24.6% (cancer survivor), respectively, vs. 12.9% (rest of the sample); *p* < 0.001). The most subjectively burdening comorbidities were reported by men identifying as “cancer survivor” and “victim” (3.5 ± 1.9 (cancer survivor) and 3.4 ± 2.0 (victim), respectively vs. 2.9 ± 1.9 (rest of the sample); *p* < 0.001). Also, men self-identifying as “cancer survivors” and “victims” reported most often frailty (44.9% (cancer survivors) and 45.6% (victim), respectively vs. 33.3% (rest of the sample); *p* < 0.001) and severe loneliness (9.6% (cancer survivors) and 26.0% (victim), respectively vs. 5.4% (rest of the sample); *p* < 0.001). The quality of life was the highest among men identifying as “cancer conquerors” (0.73 ± 0.20) and the lowest among men identifying as “cancer survivors” (0.62 ± 0.19) and “victims” (0.55 ± 0.19) (*p* < 0.001). (Table 2)

Multivariable logistic regression analyses showed an association between identification as a “patient” and an ongoing partnership and (1.47 [1.08–2.01]) as well as with a tertiary education (1.50 [1.17–1.91]). (Table 3)

**Table 3:** Factors associated with cancer-related identities in multivariable logistic regression analysis with backward elimination

	Someone who has had cancer	Patient	Cancer conqueror	Cancer survivor	Victim
	OR [95%-CI]	OR [95%-CI]	OR [95%-CI]	OR [95%-CI]	OR [95%-CI]
*Total number of burdening comorbidities SCQ [continues]*		0.93 [0.88–0.99]		1.09 [1.01–1.17]	
*Cancer a subjective comorbidities [ref. yes]*					
* no*	0.69 [0.53–0.89]	1.38 [1.05–1.82]	-	1.51 [1.11–2.08]	
*Loneliness [ref. no]*					
Yes, severe	-	-	-	-	5.09 [2.39–10.85]
Yes, mild	-	-	-	-	0.55 [0.13–2.32]
*Age at survey (years) [continues]*		-	-	-	-
* Educational level [ref. low]*					
secondary - intermediate	-	1.14 [0.84–1.55]	-	-	0.84 [0.35–2.02]
secondary - high	-	1.51 [1.10–2.09]	-	-	1.13 [0.49–2.60]
tertiary	-	1.50 [1.17–1.91]	-	-	0.23 [0.08–0.66]
*Partnership [ref. no]*					
yes	-	1.47 [1.08–2.01]	-	-	-
*Biochemical recurrence [ref. no]*					
yes during follow-up	-	-	-	1.71 [1.26–2.32]	-
yes at survey	-	-	-	1.12 [0.79–1.59]	-
*Ongoing treatment at survey [ref. no]*					
yes	0.44 [0.31–0.63]	1.89 [1.36–2.62]	-	-	-
*PHQ-2 (depression screening) [ref.* negative (< 3)*]*					
positive (≥ 3)	-	-	-	1.71 [1.20–2.45]	-
*GAD-2 (anxiety screening) [ref.* negative (< 3)*]*					
positive (≥ 3)	0.64 [0.45–0.91]	-	2.66 [1.49–4.78]	-	-
*Quality of life [continues]*	-	-	1.41[1.28–1.57]	-	-

*PHQ* Patient health questionnaire, *GAD* General anxiety disorder, *SCQ* Self-Administered Comorbidity Questionnaire, *ref* reference, *OR* odds ratio, *CI* Confidence interval

While men with no ongoing PCa treatment at survey were more likely to identify as “someone who has had cancer” (0.44 [0.31–0.63]), men with ongoing PCa treatment at the survey were more likely to identify as “patient” (1.89 [1.36–2.62]). Further, men with a biochemical recurrence during follow-up were more likely to identify as “cancer survivors” (1.71 [1.26–2.32]). Accordingly, perceiving cancer as subjective comorbidity was negatively associated with the identification as “someone who has had cancer” (0.69 [0.53–0.89]) and positively associated with the identification as “patient” (1.38 [1.05–1.82]) and “cancer survivor” (1.51 [1.11–2.08]). (Table 3)

While higher quality of life was associated with self-identification as “cancer conqueror” (1.41 [1.28–1.57]), men identifying as “cancer survivor” were more likely to record a positive screening for anxiety (1.47 [1.00-2.14]) and men identifying as “victim” were more likely to consider themselves as severely lonely (5.09 [2.39–10.85]). (Table 3)

## Discussion

Many men affected by PCa reach an age beyond 75 after initial cancer treatment. Nevertheless, the diagnosis of PCa, the treatment, and the regular follow-up may affect the self-perception and identity in these men even years after the initial diagnosis [11, 13, 25]. Further, old age is often accompanied by a general decline in fitness, health, and social interactions. However, it is unclear how these factors may be associated with specific cancer-related identities. Our data of 2,379 older men affected by PCa with a median follow-up of 17.9 years after the diagnosis of PCa and treatment with radical prostatectomy shows a gradual increase in frailty, comorbidities, and perceived loneliness with increasing age, suggesting that these factors might also interact with the general outlook on life and the perceived significance of one’s PCa. Concerning their PCa experience, most men identified as “someone who has had cancer” (45.7%), followed by “patient” (27.0%). More emotionally connoted cancer-related identities were reported by every fourth man (“cancer survivor” 15.7%, “cancer conquerors” 9.5%, “victim” 2.1%) [5, 10, 13, 16].

### Identification as a cancer survivor and victim in older men affected by prostate cancer

The prevalence of frailty, loneliness, and the number of burdening comorbidities was highest among the men identifying with more negatively connoted cancer-related identities, “cancer survivor” and “victim”. Although identification with the term “cancer survivor” was initially intended to empower individuals affected by cancer to engage more actively with their disease to improve psychological resilience, results from this analysis, as well as previous research, have shown that in men with PCa identifying as a “cancer survivor” seems to reflect a more negative, subjectively demanding cancer experience instead [5, 13, 14, 26]. Our results also show that identification as a “cancer survivor” correlates with a lower overall health status. Interestingly, this indicates that more subjective health issues do not reduce the personal significance of PCa but seem to be associated with a more negatively connoted outlook on this additional health burden.

Identification with the term “victim” was low, comparable to previous research [5, 10, 14]. Although these men represent only a minor portion of our sample, our data suggest that these men were more likely to experience severe loneliness and overall lower well-being. Interestingly, clinical factors offering an extraordinary disease course were not more common in men identifying as “victims”. This indicates that a negatively valued cancer identity among older men affected by PCa may depend mainly on subjective, not primarily, PCa-related factors and the lack of a functioning support system [8, 18, 19].

### Neutral cancer-related identities in older men affected by prostate cancer

Previously, research has shown that most men affected by PCa and treated with radical prostatectomy identify with a neutral, descriptive cancer-related identity such as “someone who has had cancer” or “patient”. These identities are primarily associated with the objective disease course and reflect the current disease status. Our results support these findings and show that these identities seem to be independent of PCa-unrelated or aging-related factors. Regarding the identification with the term “someone who has had cancer”, our results are in line with previous research, which showed that this was more common among individuals with an uneventful follow-up, which were more often cured by primary therapy and had fewer side effects [5, 10, 14]. Additionally, our data revealed that signs of anxiety were less common among these men. Overall, this suggests that many older men who have been successfully treated for PCa and subjectively do not regard this disease as a current health problem tend to perceive themselves in a very neutral way regarding their cancer experience and consider PCa as something of the past. These men also seem less likely to display signs of cancer-related distress negatively affecting well-being and probably do not require specific psycho-oncological support.

Every fourth man in our sample identified as a “patient”. Previous research has proposed that identification as a “patient” might be a sign of passiveness when dealing with cancer, which might lead to a lack of coping [14, 15]. However, it has been repeatedly shown that among men affected by PCa, the identification as patient is a neutral portrayal of an ongoing treatment without signs of a negative impact on mental health [5, 10, 26]. Our data also reflects these assumptions, showing an association between ongoing therapy and recognizing PCa as a persisting healthcare problem with the identification as a “patient”. In contrast, no association emerged with decreased overall well-being or psychological or physical fitness. Moreover, identifying as “patient” was associated with fewer burdening comorbidities and a higher likelihood of being in an intimate partnership. These results underline the assumption that in older men, identification as a PCa “patient” might not be a sign of being passively overwhelmed by cancer but rather a sign of still having the physical strength and social support to receive ongoing cancer therapy. Additionally, compared to younger individuals affected by cancer, older men with an intact social support system might be used to illnesses among their peers [3, 23]. Therefore, they might consider being a “patient” not a sign of weakness but a natural part of life. Accepting PCa as a chronic condition and identifying as a PCa “patient” might help these men to incorporate this experience into their current life situation in a neutral way without leading to additional distress.

### Active cancer identification in older men affected by prostate cancer

Every 10th man in our sample identified as a “cancer conqueror”, representing an active and positive disease engagement, embracing fighting and overcoming the struggles of a cancer diagnosis. The term “cancer conqueror” emphasizes actively overcoming PCa. Previous research has shown that an active identification with overcoming cancer seems to be associated with positive coping and improved psychological outcomes [14, 27]. In our sample, identifying as a “cancer conqueror” was more likely among men with higher overall reported well-being. These results follow previous studies suggesting that a self-confident, positive outlook toward one’s cancer experience might be associated with a better psychological adaptation [10, 11, 15, 27, 28]. Interestingly, the regression analysis did not detect any associations between the identification as a “cancer conqueror” and signs of overall higher fitness or fewer age-related health problems. This suggests that it seems advantageous to encourage men, regardless of age and age-related comorbidities, who are open to actively integrating their cancer experience into their personality in an empowering way to pursue this approach further.

### Strengths and limitations

The findings of this analysis must be considered within certain limitations. First, due to the cross-sectional design, assumptions on causality and developing a particular cancer-related identity must be treated carefully and further investigated in longitudinal studies. Based on the associations that emerged, it remains unclear whether perceived health status triggers identification with a specific cancer-related identity or whether actively identifying with a cancer-related identity leads to a particular psychological adaptation. Moreover, an unknown moderator, such as personality traits and the general outlook on life, could affect the identification with a specific cancer-related identity and subjective health status. By only including older men affected by PCa, our results do not represent other types of cancer and different life circumstances. Especially in younger individuals affected by cancer, self-perception might differ, and other disease-related factors might be of more objective and subjective importance. However, PCa is one of the most diagnosed types of cancer, and the number of older men treated for PCa with radical prostatectomy will further increase in the coming years. This makes research on these men highly relevant to understanding more of their demands from the healthcare community. Lastly, it should be noted that this study was conducted in Germany. The translation of terms such as “survivor” or “conqueror,” which are associated with distinct cultural interpretations, may result in alterations to the nuances of meaning, and therefore the results of this study may not be applicable in other cultural contexts. Further research is necessary to address how diverse cultural and linguistic backgrounds might influence the understanding and usage of these terms among individuals affected by cancer. Lastly, it must be pointed out that 31% of contacted men did not return the questioner. Therefore, we cannot conclude whether these men differ in regard to their psychosocial status compared to the men that returned the questionnaire. This limits the generalizability of our date. Nevertheless, the median age and clinical characteristics, such as cancer stage and prostate cancer therapy, did not differ significantly between those who responded and those who did not.

## Conclusion

In conclusion, most men aged beyond 75 after being affected by PCa and treated with radical prostatectomy consider PCa as something of the past and do not consider it a subjective, ongoing health problem. These men identify with neutral terms such as “someone who has had cancer”. Moreover, it is common among men who require continuous PCa therapy to identify as a “patient”, which should be considered a neutral self-description, not necessarily implying an unmet need for psycho-oncological support. Negatively connoted PCa-related identities are associated with distinct characteristics, which are only partially related to objective clinical factors but rather reflect the subjective disease burden, overall health status, the presence of a social support system, and overall well-being. Some of these factors also seem to become more evident with age and might deepen a negative PCa-related identity in older men. Therefore, it should be recommended that healthcare professionals facilitate reflection among men affected by PCa to identify how they perceive themselves with regard to their disease. This will enable healthcare professionals to identify those who require further assistance and ensure that these men are addressed adequately. Furthermore, in older men, the signs of aging, such as frailty and loneliness, should be assessed regularly, as they appear to be associated with overall perceived health status and psychological adaptation in men affected by PCa.

## Data Availability

No datasets were generated or analysed during the current study.
